# Methods and Applications in Respiratory Physiology: Respiratory Mechanics, Drive and Muscle Function in Neuromuscular and Chest Wall Disorders

**DOI:** 10.3389/fphys.2022.838414

**Published:** 2022-06-14

**Authors:** Nina Patel, Kelvin Chong, Ahmet Baydur

**Affiliations:** ^1^ Dornsife College of Letters, Arts and Sciences, Division of Pulmonary, Critical Care and Sleep Medicine, Keck School of Medicine and University of Southern California, Los Angeles, CA, United States

**Keywords:** neuromuscular disease, respiratory mechanics, dyspnea, control of ventilation, expiratory flow limitation, diaphragmatic fatigue, gas exchange, respiratory pressures

## Abstract

Individuals with neuromuscular and chest wall disorders experience respiratory muscle weakness, reduced lung volume and increases in respiratory elastance and resistance which lead to increase in work of breathing, impaired gas exchange and respiratory pump failure. Recently developed methods to assess respiratory muscle weakness, mechanics and movement supplement traditionally employed spirometry and methods to evaluate gas exchange. These include recording postural change in vital capacity, respiratory pressures (mouth and sniff), electromyography and ultrasound evaluation of diaphragmatic thickness and excursions. In this review, we highlight key aspects of the pathophysiology of these conditions as they impact the patient and describe measures to evaluate respiratory dysfunction. We discuss potential areas of physiologic investigation in the evaluation of respiratory aspects of these disorders.

## 1 Introduction

Neuromuscular and chest wall disorders impact breathing in a manner different from injury to the lungs. Respiratory muscle weakness, reduced lung volume and increases in respiratory elastance and resistance lead to increase in work of breathing, respiratory pump failure and impaired gas exchange (Campbell, 1965). The main objective of this review is to highlight key aspects of the pathophysiology of these conditions as they impact the patient and describe recently developed measures to evaluate respiratory dysfunction. We describe key aspects of normal respiratory muscle structure and function, how they are affected in neuromuscular disorders (NMDs), current methods of functional evaluation, and recent advances in their assessment. We finish with suggestions for potential applications of newer techniques that may be considered for their evaluation. We focus primarily on the pathophysiology of NMDs as they impact adults but will refer to changes in children where relevant.

## 2 Pathophysiology

### 2.1 Normal Respiratory Muscle Function

The respiratory system functions to secure gas exchange between ambient air and blood to maintain arterial blood gas pressures within certain acceptable values. It consists of the lungs, chest wall (including respiratory muscles), controllers of breathing, and spinal cord and peripheral nerves that communicate with the respiratory system. Neuromuscular and chest wall disorders affect these structures in varied ways, depending on the age of onset, distribution of neuro/myopathic involvement and rate of progression of the disorder.

**FIGURE 1:**
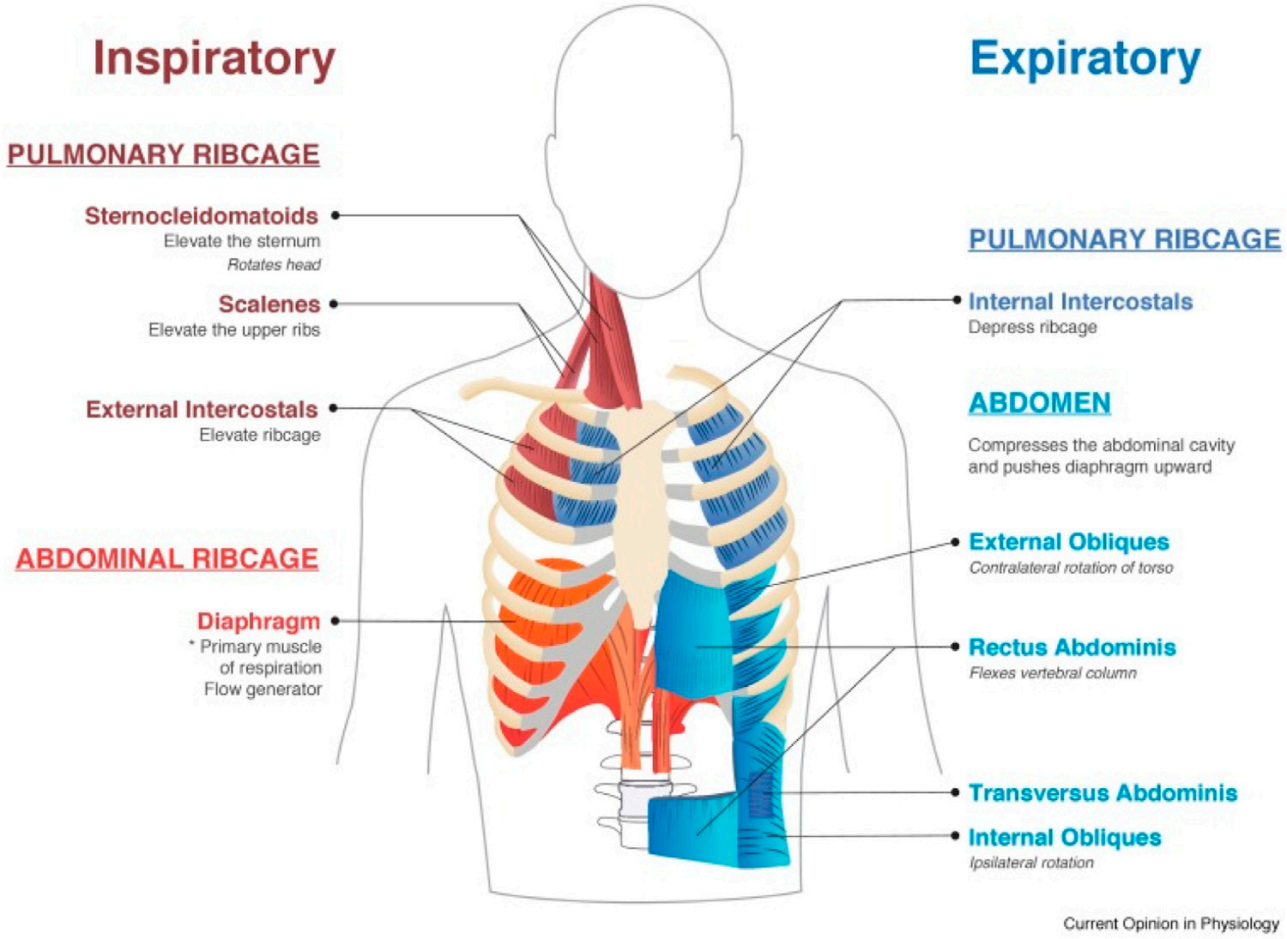
Muscles involved in normal inspiration and expiration. From Welch et al., 2019.

Respiratory muscles are classified into those with inspiratory and expiratory function (Figure 1) (Campbell, 1965; Benditt, 2019; Welch et al., 2019). In healthy individuals during quiet breathing, the diaphragm is the only active inspiratory muscle; expiration is achieved by passive relaxation of the lungs. During increased demand such as exercise, other muscles become selectively active depending on the relative increase in inspiratory or expiratory requirements, or both. Upper airway dilator muscles maintain patency of the pharynx and larynx, preventing upper airway occlusion, an event occuring frequently with bulbar dysfunction and exacerbated during sleep-disordered breathing.

### 2.2 Diaphragm

**FIGURE 2:**
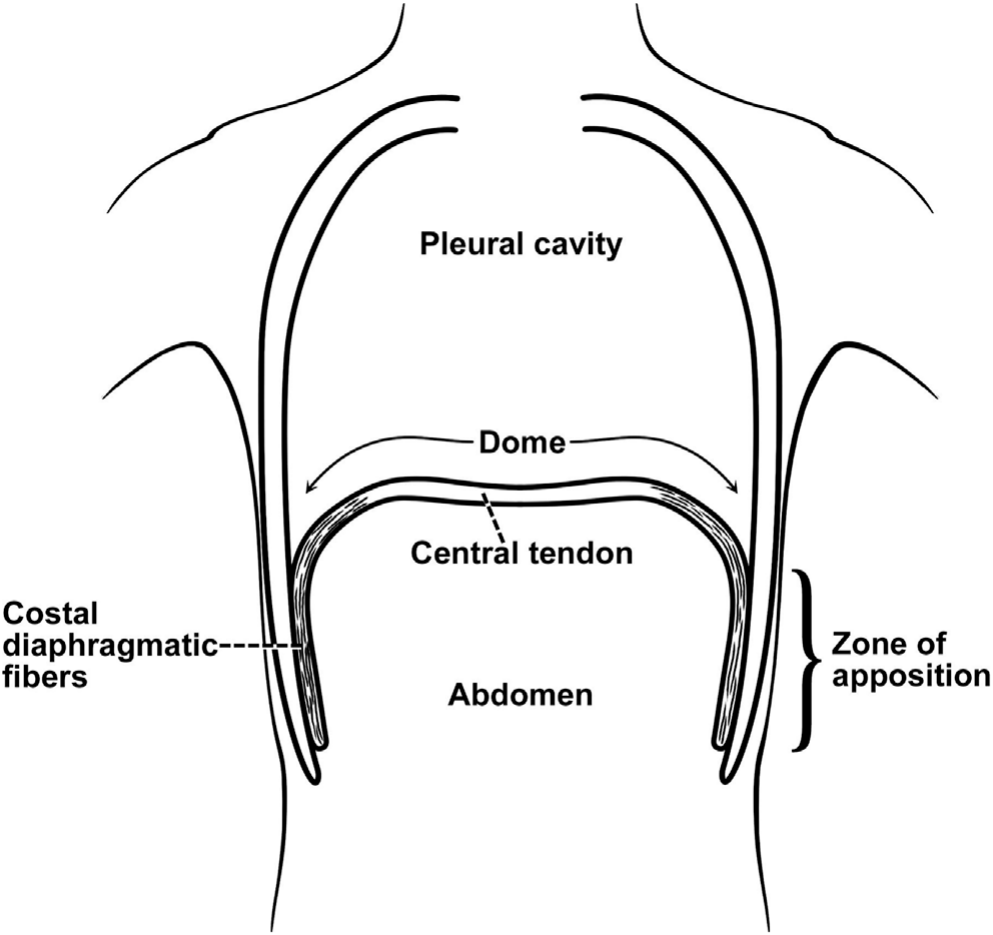
Zone of apposition of the diaphragm. The muscle fibers of the diaphragm originate from the circumference of the thoracic outlet, in particular the lower ribs, and converge to insert into a central tendon). From their origins on the lower ribs the fibers run mainly cranially and are directly parallel (apposed) to the inner aspect of the lower rib cage, constituting the “zone of apposition.” From (Troyer and Wilson, 2016).

The diaphragm is a thin sheet of muscle acting as a piston, decreasing intrathoracic pressure and drawing air into the lungs. It enables the ribs to move up and outwards (“bucket handle” action), increasing their transverse span (Benditt, 2019). It is configured like an elliptical cylindroid with a dome cap. The cylindrical portion of the diaphragm shortens during inspiration while the dome changes little. Two components counterbalance the inspiratory action: the appositional and insertional components. The zone of apposition is represented by the muscular portion inserted into and parallel to posterolateral aspect of the abdominal wall (Figure 2). The increase in abdominal pressure on the lower rib cage constitutes the appositional portion while the action of the diaphragm in relation to the rib cage constitutes the insertion portion (Mead, 1979; Loring and Mead, 1982; Troyer and Wilson, 2016). During tidal breathing in upright position the diaphragm and intercostal muscles contribute to 70% and 30% of the Vt, respectively (Druz and Sharp, 1981). In supine posture the contribution of diaphragm increases to 90% (Mognoni et al., 1969; Druz and Sharp, 1981), predisposing to orthopnea. The diaphragm generates only 10–20% of its maximum force generation during quiet breathing (Benditt, 2019; Welch et al., 2019).

### 2.3 Intercostal muscles

Intercostal muscles span the intercostal space as two thin layers. The inner intercostal fibers run in the caudal-dorsal direction from the sternocostal junction to near the tubercles of the ribs (De Troyer and Estenne, 1984). Fibers of the external intercostals run caudal-ventrally, extending from the tubercles of the ribs to the costochondral junction.

The functions of intercostal muscles have been controversial, particularly because they cannot be activated individually and are inaccessible. De Troyer et al. (De Troyer and Estenne, 1984) found that external intercostal muscles possess a substantial inspiratory mechanical advantage that decreases in the caudal and ventral directions until it is reversed into an expiratory mechanical advantage. The internal intercostals primarily exhibit an expiratory mechanical advantage (Benditt, 2019) while the intertriginous portion of the internal (parasternal) intercostals also possess an inspiratory advantage (De Troyer and Estenne, 1984; Benditt, 2019). External intercostal muscles additionally stabilize (stiffen) the rib cage, minimizing its inward collapse during inspiration while maintaining ventilation-perfusion relationships (Roussos).

### 2.4 Accessory Inspiratory Muscles

The accessory inspiratory muscles consist of the sternocleidomastoids (SCM), scalenes, pectoralis major and minor, and inferior fibers of serratus anterior and latissimus dorsi. The sternocleidomastoids elevate the clavicles and first rib, reducing pleural pressure and cause the abdomen and lateral ribcage to expand outward (De Troyer and Estenne, 1984; Roussos). They become active at high tidal volume generation, such as during exercise (Roussos). Sternocleidomastoid activity increases during respiratory distress from any cause, but has been mainly documented in patients with chronic obstructive pulmonary disease (COPD) (Sarkar et al., 2019): more than a 5 mm upward movement of the clavicle is associated with severe airflow limitation, reflected by an FEV_1_ of 0.6 L or less (Sarkar et al., 2019). The scalenes arise from the transverse processes of the lower cervical vertebra and insert into the first 2 ribs. They contract under increased stress and metabolic demand, expanding the upper rib cage to augment tidal breathing.

Bastir et al. (Bastir et al., 2017) showed that expansion and contraction (kinematics) of the pulmonary and diaphragmatic parts of the thorax differed in their modes of shape change during breathing while the degree of shape change was similar in both compartments. The diaphragmatic part exhibited expansion more than the pulmonary part, therefore the upper thorax has to undergo greater deformation to expand to the same degree as the lower thorax. This has important implications with regard to respiratory muscles weakened at different times. For example, in ascending paralysis, the lower rib cage is likely to exhibit inward retraction and collapse as the intercostals weaken, while the diaphragm and accessory neck muscles assume increasing inspiratory activity. Thus, variability in regional lung expansion, ventilation-perfusion matching and gas exchange ensue depending on which thoracic muscles are impaired and the time over which these changes evolve (Roussos).

### 2.5 Abdominal Muscles

Four primary abdominal muscles augment expiratory force—the rectus abdominus, transverse abdominus, internal abdominal oblique and external abdominal oblique (Welch et al., 2019; Roussos). They increase intraabdominal pressure by pulling the abdominal wall inwards and displacing the diaphragm up into the thoracic cavity. In addition they lower the ribs, pulling them medially thereby deflating the ribcage, key during forced expiration such as coughing.

Abdominal muscles indirectly aid with inspiration. They compress the lungs to below their normal end-expiratory volume, storing elastic energy in the chest wall during expiration, facilitating passive inspiration. This action increases the curvature of the diaphragm and its force generation (based on Laplace’s Law) (Roussos).

### 2.6 Upper Airway Dilator Muscles

Dilator muscles of the pharynx and larynx facilitate air flow by minimizing upper airway resistance during inspiration, an important function compromised in patients with bulbar dysfunction, and more so in individuals with obstructive sleep apnea (Oliven et al., 2018). Airway patency is maintained by coordinated co-activation of inspiratory and upper airway muscles, including the genioglossus, and preventing collapse of pharyngeal soft tissues (Van de Graaff et al., 1984; Kobayashi et al., 1996; Malhotra et al., 2000; Eckert et al., 2007; Fauroux and Khirani, 2014; Oliven et al., 2018). Posterior movement of the hyoid during inspiration can increase airway resistance and limit airflow, especially during sleep, an action opposed by contraction of the sternothyroid, thyrohyoid, sternohyoid, and geniohyoid muscles to preserve upper airway patency (Van de Graaff et al., 1984).

## 3 Reductions in Respiratory System Compliance and Volumes

**FIGURE 3:**
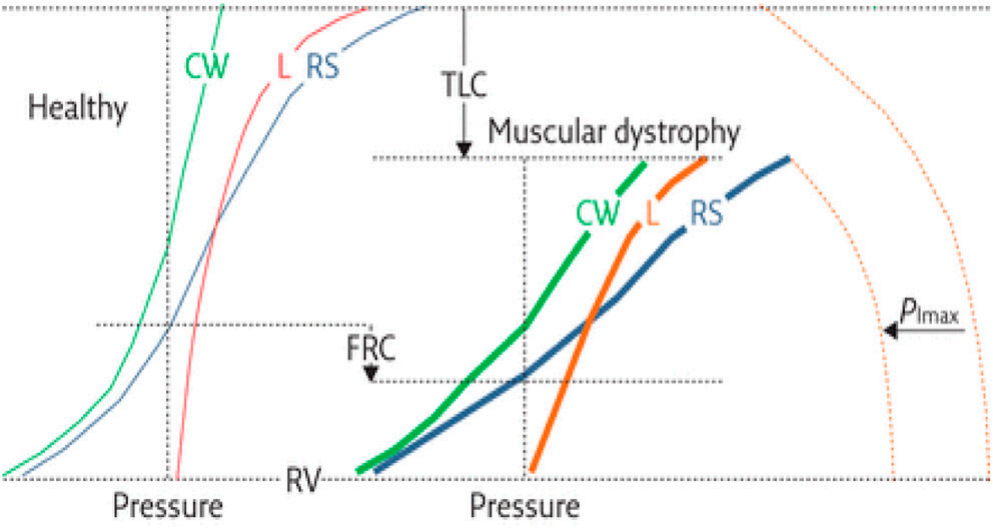
Respiratory mechanics in individual with muscular dystrophy compared to that of a healthy individual. Shown are the presumed pressure–volume curves of the respiratory system (RS, blue curves) and its two components, the chest wall (CW, green curves) and lung (L, red curves), for healthy subjects (thin curves on the left panel) and patients with muscular dystrophy (thick curves on the right panel). The maximal inspiratory pressure (PImax) is also shown (dashed curves). Key features include 1) decreased total lung capacity (TLC); 2) decreased compliance of chest wall (CCW), lungs (CL) and respiratory system, a consequence of reduced thoracic volume (slopes of the corresponding pressure–volume curves); 3) reduced PImax; 4) decreased inspiratory capacity (IC = TLC−FRC); and 5) reduced expiratory reserve volume (ERV = FRC−RV). Functional residual capacity (FRC) may be reduced or even normal. Residual volume (RV) is usually preserved. PImax represents the force of inspiratory muscles, while the volume variations are the resulting action of their contraction. The compliance of the respiratory system is decreased because of **(A)** lung atelectasis and fibrosis, the former a consequence of hypoventilation, and the latter from recurrent aspirations; and **(B)** scoliosis, induced by assymmetric involvement of trunk muscles. Figure caption modified from (Lo Mauro and Aliverti, 2016).

**FIGURE 4:**
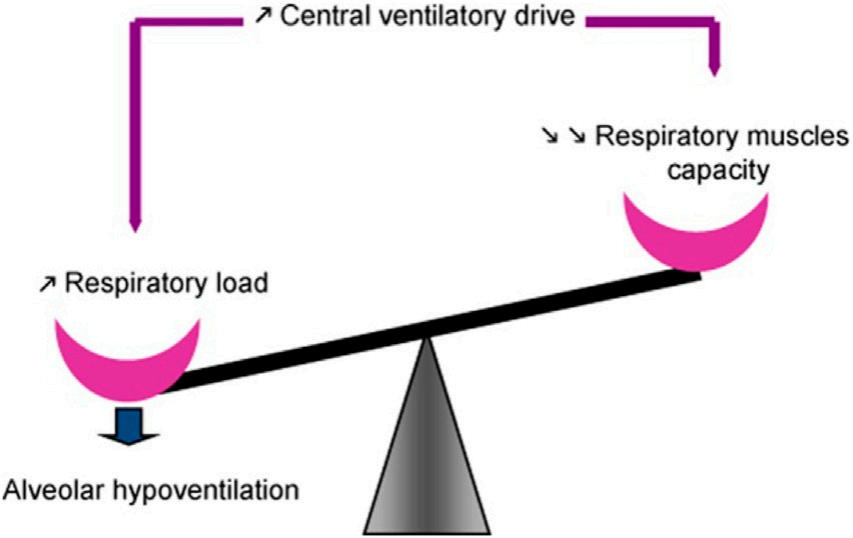
Maintenance of spontaneous ventilation depends on balance between function of the respiratory muscles on one side and the respiratory load, determined by respiratory mechanics, on the other. Central respiratory drive is regulated such that work of breathing is minimized by adjusting tidal volume and respiratory rate to compensate for the load. Alveolar hypoventilation ensues when the respiratory load exceeds the ability of the central drive and respiratory muscles to maintain adequate gas exchange, often associated with respiratory muscle fatigue. From (Fauroux and Khirani, 2014).

Patients with NMD exhibit reduced vital capacity (Fauroux and Khirani, 2014; Lo Mauro and Aliverti, 2016) and respiratory (lung + chest wall) compliance (increase in respiratory elastance) (Figure 3), in part due to alveolar collapse and, when present, scoliosis. Recurrent aspiration contributes to further reduction in lung compliance with loss of aerated alveolar volume, as seen with acute respiratory distress syndrome, and a serious complication of aspiration (Zaloga, 2002). Upper airway collapse from bulbar dysfunction further increases resistance to breathing (Benditt, 2019). Such changes in compliance contribute to increase in work of breathing. Work of breathing is kept at the lowest level by adjusting tidal volume and respiratory rate to compensate for the load. Alveolar hypoventilation results from the respiratory load exceeding the ability of the central drive and respiratory muscles to maintain adequate gas exchange (Figure 4).

In supine posture gravitational pressure from abdominal contents impairs tidal excursions of the diaphragm. In most NMDs a decrease in VC upon assuming supine posture (by 25% or more) is a sensitive index of diaphragmatic weakness (Fromageot et al., 2001; Lechtzin et al., 2002; Prigent et al., 2012; Chen et al., 2013). In this connection, in the upright position, application of an abdominal wrap below the lower rib margin displaces the diaphragm cephalad, lengthening its fibers, increasing its force generation and reducing dyspnea, a technique widely adopted by poliomyelitis patients during the epidemic of the 1940s and 1950s to improve activities of daily living and quality of life (Bach, 2017). Individuals with high cervical cord injury and others with weak abdominal musculature also find abdominal wraps to be helpful with breathing (Goldman et al., 1986; McCool et al., 1986; Scott et al., 1993; Berlowitz et al., 2016). Their VC increases as diaphragmatic fibers lengthen with increase in force generation and reversal of platypnea (Fugl-Meyer and Grimby, 1984; Baydur et al., 2001; Terson de Paleville et al., 2014). This finding is in line with studies in able-bodied individuals in which maximal force generation of the transdiaphragmatic pressure (Pdi) decreases as lung volume increases (Beck et al., 1998) and would explain the increase in diaphragmatic strength in the supine spinal cord-injured patient.

## 4 Effects of Obesity

Similar to able-bodied people, individuals with NMD are at risk for developing chronic diseases resulting from obesity and a sedentary lifestyle. Aitkens et al. (Aitkens et al., 2005) found that 55% of their NMD patients met criteria for metabolic syndrome. Bauman et al. (Bauman et al., 1992) determined that cardiovascular disease was the leading cause of death in individuals with spinal cord injury and occurred at a younger age than in able-bodied persons.

Changes in respiratory mechanics occurring in NMD are augmented with obesity. Oxygen cost and work of breathing are increased to overcome respiratory resistance or inertance (Dempsey et al., 1966; Sharp et al., 1986; Kress et al., 1999). Despite the increase in gravitational load of the chest wall in the supine position, functional residual capacity (FRC) does not fall below seated values in obese subjects with reduced FRC when seated. In supine position inspiratory muscle activity maintains dynamic hyperinflation above relaxation volume presumably because of greater muscle length and force generation (Yap et al., 1995).

In a retrospective study of 34 patients with Duchenne muscular dystrophy (DMD, mean age 13.7 years) Chew et al. (Chew et al., 2016) found that BMI was positively related to FVC, contrary to what one might expect in obese individuals (Sharp et al., 1964; Thomas et al., 1989; Salome et al., 2010). The observed association may have been the result of corticosteroid therapy resulting in greater muscle mass and strength, although the study did not take into account changes in muscle and fat mass, edema or growth, also known to occur in DMD (Durnin and Rahaman, 1967; Stanbury and Graham, 1998; Pessolano et al., 2003).

## 5 Control of Ventilation and its Relation to Respiratory Load Compensation

The link between the nervous system and respiratory muscles are the motor neurons located in the brain stem. The ventral medulla regulates exhalation while the dorsal medulla is responsible for inhalation. Communication between the two parts of the medulla is through integration of input from peripheral and central respiratory muscles to generate a cyclical respiratory rhythm. The pontine group allows for regulation of the medullary signals to ensure smooth inspiration and expiration transition. Central chemoreceptors located within the medulla and retrotrapezoid nucleus to sense pH changes related to carbon dioxide concentrations in the cerebrospinal fluid (Panitch, 2009). Peripheral chemoreceptors (carotid and aortic bodies) respond primarily to changes in arterial oxygen tension.

As early as the poliomyelitis epidemic (Whittenberger and Sarnoff, 1950; Plum and Swanson, 1958), changes in central control of ventilation have been described in various NMDs (Kilburn et al., 1959; Carroll et al., 1976; Rosenow and Engel, 1978a; Borel et al., 1993; Rialp et al., 2013). A blunting of the ventilatory response to hypercapnia implies a decrease in chemoreceptor sensitivity; however, in the presence of diffuse muscle weakness and abnormal respiratory mechanics, determining the exact contribution of the medullary motoneurons to a decrease in ventilatory response can be a challenge (Borel et al., 1993; Rialp et al., 2013). Measurement of occlusion pressure (P_0.1_) provides a more reliable assessment of central drive as it is not influenced by change in lung volume and flow resistance (Whitelaw et al., 1975). Indeed, some studies have demonstrated normal or increased P_0.1_ (McCool et al., 1988; Baydur, 1991), indicating that central drive is intact. In many cases, sleep-disordered breathing contributes to nocturnal hypoventilation even as the patient is able to maintain normal daytime gas exchange (Aboussouan, 2015; Fermin et al., 2016). An inspiratory vital capacity of less than 60% predicts sleep disordered breathing in children and adolescents with NMD [59 Fermin]. Overnight polysomnography confirms presence of sleep-disordered breathing. Sleep abnormalities of central origin can be seen in myotonic dystrophy, which may be separate from the muscular deficit. We refer the reader to excellent reviews on sleep-disordered breathing for in-depth discussions of their diagnosis and management (Aboussouan, 2015; Fermin et al., 2016).

Axen (Axen, 1982) described variable changes in ventilatory control (tidal volume and inspiratory duration) in cervical cord-injured men in response to elastic and resistive loads, assuming identical respiratory muscle pressure (Pmus) wave forms in the unloaded and loaded states. Afferent pathways from the mouth, lung, and/or diaphragm modulated the phrenic discharge during the first loaded breath. In chest wall disorders such as kyphoscoliosis (KS), respiratory elastance and resistance are increased, and breathing pattern is rapid and shallow, resulting in defense of tidal volume (Vt) in the face of inspiratory resistive loading. Baydur and Carlson (Baydur and Carlson, 1996) computed passive elastance (Ers) and active elastance and resistance (E′rs and R′rs, respectively) in anesthetized patients according to previously described techniques (Behrakis et al., 1983; Baydur et al., 1989). The difference between passive and active respiratory elastance represents changes in thoracic mechanical properties (stiffening) related to chest wall distortion during added loads. With resistive loading, driving pressure and inspiratory time were prolonged compared to healthy subjects, while percent reduction in Vt and minute ventilation was less in KS. Increased intrinsic impedance, Pmus, and differences in changes in neural timing in anesthetized kyphoscoliotics contributed to modestly greater Vt defense, compared to that of anesthetized subjects free of cardiorespiratory disease.

### 5.1 Elastic Load Compensation

If someone were to hold a 20-lb weight with arm outstretched and had a 1-lb weight added to the load, they are not likely to feel the added weight as afferent sensory receptors adapt to the high muscle tension; by contrast, they are more likely to feel the addition of a 10-lb weight which noticeably adds to the tension. A similar analogy can be applied in individuals with increased elastic respiratory loads. To evaluate the effects of abnormal respiratory mechanics and neuromuscular drive on the various components of elastic load compensation, Baydur et al. (Baydur et al., 1989) studied anesthetized patients with KS whose mean passive and active respiratory elastance, active respiratory resistance, and peak inspiratory occlusion pressure were, respectively, 89%, 84%, 100%, and 37% greater, and inspiratory duration (Ti) 13% less than corresponding values in anesthetized control subjects. The increased intrinsic elastance and resistance and decreased Ti contributed to Vt defense in KS in the absence of vagal modulation. Characteristics of elastic load compensation in anesthetized KS patients are nevertheless similar to those of anesthetized normal subjects.

### 5.2 Post-Inspiratory Diaphragmatic Braking Activity

The diaphragm has been shown to exhibit post-inspiratory activity during passive expiration (pliometric activity) (Muller et al., 1979; Muller et al., 1980; Zin et al., 1983; Baydur, 1992; Easton et al., 1999) resulting in preservation of lung volume. Such activity may be of benefit in patients with chest wall disorders in preventing airway collapse (Muller et al., 1980; Baydur, 1992). Absence of pliometric activity may result in reduction in lung volume and atelectasis.

## 6 Dyspnea in Patients With Neuromuscular Disease: Concept of Air Hunger Vs. Perception of Respiratory Work and Effort

The sensation of an urge to breathe is referred to as “air hunger”, for example, when it develops during a long breath hold (Banzett et al., 2021) it occurs when urgent homeostatic needs to maintain gas exchange are not met. Stimuli that increase air hunger include hypercapnia, hypoxia, exercise, and acidosis; tidal expansion of the lungs (spontaneous or assisted) reduces air hunger. As such, it is not affected by respiratory muscle activity and is thought to be regulated by central chemoreceptors in the brain stem (Banzett et al., 1990). By contrast, when respiratory muscle fatigue or an increase in breathing load (such as occurs with pneumonia) calls for increase in muscle contraction, a sensation of increased work or effort is described; it likely arises from respiratory muscle afferents or from the brainstem or cerebral cortex.

A striking example of these events is that of a chronically over-ventilated muscular dystrophy patient whose PaCO_2_ has gradually decreased to the 20–30 mm Hg range (a common issue as patients feels better with sensation of increase in chest wall expansion with increase in Vt. Patients receiving nocturnal ventilation often maintain a lower PCO_2_ during the following day. Possible explanations include: 1) Reducing daytime work of breathing by resting respiratory muscles at night, and 2) CO_2_ elimination at night promotes bicarbonate excretion with a lower set point for ventilatory control as metabolic alkalosis is corrected. Allowing the PCO_2_ to increase by intermittently reducing the delivered Vt (even by 30–50 ml at a time) or respiratory rate become uncomfortable for the patient, increasing demand that ventilator settings be restored to their original state (personal observation). These symptoms suggest a re-set of the threshold for CO_2_ sensitivity such that even a slight rise in PCO_2_ induces air hunger despite (or because of) the chronic hypocapnia. Coughing induced by airway irritation can aggravate the sensation of air hunger and might be vagally mediated but central mechanisms cannot be ruled out (Nishino et al., 2008).

By contrast, individuals will describe a sensation of feeling “hard to breathe” when experiencing a respiratory infection because of alterations in respiratory compliance and resistance (with little or no change in blood gases). Changing ventilator settings by increasing the Vt or respiratory rate relieves their symptoms, but at the cost of inducing a chronic respiratory alkalosis. The resulting reduction in plasma bicarbonate stores increases vulnerability to metabolic acidosis resulting from sepsis, shock and other causes.

## 7 Respiratory Muscle Fatigue

Respiratory muscle fatigue is defined by loss in capacity for developing force against a constant load (Roussos and Koutsoukou, 2003). The higher the diaphragmatic force generation is as a function of maximal pressure the diaphragm can sustain, the greater the chance the diaphragm is likely to fatigue. Multiple factors contribute depending on how the NMD affects the central nervous system, transmission of signals between the CNS and muscle (central fatigue) or even the muscle itself (peripheral fatigue). Central muscle fatigue results from a deficit in the capacity to recruit all muscle units, while peripheral fatigue is due to a failure of muscle fibers to respond maximally during full activation (Edwards, 1979). Weakening of respiratory muscles produces paradoxical breathing (asynchronous movement) between abdomen and ribcage (Troyer and Wilson, 2016). As the diaphragm fatigues, inspiratory action assumed by the accessory muscles of the neck results in inward retraction of the ribcage and outward displacement of the abdominal wall (Benditt, 2019).

Measurement of transdiaphragmatic pressure (Pdi) (using esophageal and gastric balloons) is a more precise means of recording diaphragmatic force generation (Pdi) and endurance (Roussos et al., 1979a; Bellemare and Grassino, 1982). It is, however, invasive and requires coaching for naïve individuals, and therefore primarily used in research. The individual is asked to breathe through a series of inspiratory flow resistors to achieve a target pressure on an oscilloscope or computer screen. From these measurements, the tension-time index [TTdi = (Pdi/Pdimax) (Ti/Ttot), where Ti is the inspiratory time and Ttot is the duration of respiratory cycle] can be computed. TTdi values of >0.18 are associated with respiratory muscle fatigue and reduced endurance (Roussos et al., 1979a; Bellemare and Grassino, 1982). The technique is capable of producing inspiratory pressures generated by rib cage muscles in the absence of diaphragmatic contribution. A potential clinical application of Pdi in individuals with NMD is to determine which inspiratory muscles contribute to respiratory insufficiency and cough impairment (as a result of loss of lung volume and elastic recoil): Alternating amplitude of Pdi and gastric pressure (Pga) indicates recruitment and derecruitment of different groups of inspiratory muscles (Roussos et al., 1979a).

A non-invasive alternative is to measure the tension-time index of the inspiratory muscles as a whole (TTlim) during mouth breathing without the use of esophageal and gastric balloons (Ramonatxo et al., 1995). An increase in mean inspiratory pressure in relation to the maximal inspiratory pressure produces an increase in TTlim. Another noninvasive tool analogous to the TTdi is the breathing intolerance index (BIT), used to assess the ability of patients with NMD and other conditions to wean off ventilation (Koga et al., 2206). It makes use of the relation of breathing pattern to vital capacity (VC) [(Vt/VC) (Ti/Ttot)]. In this analysis, lung volumes replace respiratory muscle pressures so that the ratios comprising BIT can be affected by lung and chest wall mechanics in addition to respiratory muscle strength. Later, Baydur and Chen (Baydur and Chen, 2013) found BIT in patients with obesity (a chest wall disorder) tended to be higher than in healthy controls in contrast to patients with chronic obstructive pulmonary disease.

Sarmento and colleagues (Sarmento et al., 2021) found that *1)* inspiratory rib cage muscles (sternocleidomastoids, scalenes and parasternals) differed in their responses to fatigue and recovery, as reflected by changes in spectral surface EMG variables, *2)* loss of mechanical power in rib cage muscles resulted from reduced shortening velocity, and *3)* relaxation properties recovered better from fatigue than do contractile characteristics. Recovery of fatigue varied, with median frequency returning to pre-fatigue values faster than the high/low (H/L) frequency spectrum suggesting that mechanisms of fatigue differed depending on the specific inspiratory muscle. Furthermore, changes in relaxation rates were strongly associated with the H/L frequency spectrum and predicted inspiratory ribcage muscle recovery. Again, the rate of progression of respiratory impairment (or stabilization) will vary depending on which groups of muscles are involved and their characteristics of fatigability. In addition to twitch characteristics assessed by EMG (Bellemare and Roussos, 1995), muscle fatigue characteristics may be presaged by periodic analysis of their H/L frequency spectrum.

## 8 Physiology of Cough: Neuromechanical Coupling

Patients with NMD experience difficulty clearing airway secretions due to ineffective cough (Perrin et al., 2204; Yang and Finkel, 2010; Sahni and Wolfe, 2018; Allen, 2010). For an effective cough, one needs to take a deep breath, while the glottis closes to increase intrathoracic pressure, followed by its opening explosively in conjunction with abdominal contraction to expel air. Peak cough flows below 160 L/min are considered ineffective (Bach, 2017). Glottic and bulbar dysfunction lead to accumulation of saliva in the valleculae and pyriform sinuses (Silbergleit et al., 1991; Sonies and Dalakas, 1991). As a result, ineffective airway clearance predispose to recurrent pneumonia a major cause of mortality and morbidity (Chatwin et al., 2018).

The cough reflex is chiefly mediated by vagal afferent nerve fibers innervating the larynx, large airways and parenchyma; its components comprise both myelinated (rapidly and slowly adapting receptors) and unmyelinated rapidly adapting and noniceptive pulmonary fiber (C-fiber) receptors (Widdicombe, 1954; Paintal et al., 1986). The latter fibers assume a major role in cough and related reflexes. Deep inspiration stimulates airway irritant receptors, therefore an impaired inspiratory effort will diminish triggers to cough as well as the effort. Other neural pathways regulating the cough reflex include the glossopharyngeal and phrenic nerves which have many sensory as well as motor fibers. Efferent pathways include (again) the *1)* vagus (motor nerve for the muscles of the pharynx and larynx); *2)* the spinal motor nerves of which the thoracic supply the intercostal muscles and the lumbar nerves the abdominal muscles; and *3)* the trigeminal, facial, hypoglossal and accessory nerves innervating the upper airway and accessory muscles, called into play during cough (Bouros and Green, 1971).

Patients with spinal cord injury exhibit respiratory muscle dysfunction to the extent reflected by the level of cord injury (Ledsome and Sharp, 1981; Fugl-Meyer and Grimby, 1984; Baydur et al., 2001). High cord injuries leave intact residual action by the diaphragm and accessory muscles with cough generation dependent on high lung elastic recoil. Even with such injuries, however, active expiration in quadriplegic individuals can be augmented by contraction of the clavicular portion of the pectoralis major (innervated by the 5th through 7th cervical segments) which displaces the manubrium and upper ribs downward, contracting the upper rib cage (De Troyer et al., 1986), an important consideration for cough generation. By contrast, the lower rib cage expanded, at least in early inspiration, as a result of increase in abdominal pressure. Estenne et al. (Estenne et al., 1994) later showed that despite weak expiratory muscles, quadriplegic individuals were able to enhance their cough ability as a result of dynamic compression of tracheal and large airways enabling marked increase in expiratory flow, even though peak pleural pressures were 74–92% less than in normal subjects.

## 9 Respiratory Failure

Respiratory failure develops from impairment of ventilation and gas exchange due to lung or chest wall dysfunction. Increases in respiratory elastance and resistance due to lung volume reduction and thoracic cage distortion lead to increase in work of breathing and respiratory muscle fatigue. Typically, arterial oxygen tensions lower than 8.0 kPa and arterial carbon dioxide tensions above 6.0 kPa define respiratory failure. These values should serve as guides to identifying respiratory failure, not as rigid cut-offs (Roussos and Koutsoukou, 2003; Welch et al., 2019).

There are two types of respiratory failure—hypoxemia with and without hypercapnia (Roussos and Koutsoukou, 2003). The former is a result of inadequate ventilation due to reduced neural drive and muscular power as occurs with progressive NMDs. Hypercapnic failure reflects increase in physiological dead space, manifested during rapid, shallowing breathing due to abnormal chest wall mechanics (Welch et al., 2019). Hypoxemia without hypercapnia results from impaired oxygen transfer, a consequence of atelectasis and chest wall distortion (Campbell, 1965; Welch et al., 2019). Microatelectasis results in an increase in the alveolar-arterial oxygen difference (AaDO2), reflected by a normal PaO_2_, sometimes in conjunction with a reduced PaCO_2_ while breathing room air. In individuals with sleep apnea, loss of upper airway muscle tone and collapse coupled with weakening thoracic cage muscles lead to impaired gas exchange, exaggerated during REM sleep (Benditt, 2019). As sleep disordered breathing worsens, hypercapnia persists throughout day and night.

Acute decompensation may result from aspiration due to bulbar dysfunction-induced dysphagia or gastroesophageal reflux (Allen, 2010; Wijdicks, 2017; Welch et al., 2019), requiring assisted ventilation. In this connection, long-term ventilatory support can result in further weakening of the diaphragm due to muscle atrophy (Roussos et al., 1979b; Jaber et al., 2011) and passive increase in end-expiratory volume by application of positive end-expiratory pressure (PEEP) (Jansen et al., 2021).

## 10 Evaluation of Respiratory Muscle Function and Physiologic Changes in NMD

### 10.1 Electrophysiologic Techniques

Relating the electrical activity of the diaphragm to its force generation during quiet breathing or maximal respiratory efforts is used to assess diaphragmatic weakness and its propensity to fatigue under conditions of increased load (Bellemare and Grassino, 1982; Bellemare and Roussos, 1995; Sarmento et al., 2021). Assessing the ratio of diaphragm compound muscle action potential (CMAP) amplitude to transdiaphragmatic twitch pressure is also used to distinguish between defects in neuromuscular transmission defects (when both quantities decrease) and defects in muscle contractility (when twitch pressure is decreased in presence of a normal CMAP) (Aldrich et al., 1986).

### 10.2 Respiratory Pressures

Traditionally, respiratory muscle strength has been measured with maximal inspiratory and expiratory mouth pressures (Black and Hyatt, 1971; Leech et al., 1983; Vincken et al., 1987). Padkao and Boonla (Padkao and Boonla, 2020) showed that Pimax and Pemax were significantly related to middle and lower thoracic wall expansion, suggesting that respiratory muscle strength was more closely associated with chest wall expansion and diaphragmatic descent than with expiratory muscle strength. If thoracic cage muscles are weakened by disease, rib cage expansion is impaired and the rib cage fails to expand, these maneuvers are, however, difficult to perform by patients with weak muscles, as they require cooperation and coordination, Pimax (MIP) and Pemax (MEP) are ideally generated at residual volume and total capacity, respectively, and need to be maintained for 4 s, a challenge even for able-bodied individuals (Aldrich and Spiro, 1995).

As alluded to earlier, respiratory impairment depends on the pattern of muscle and/or nerve involvement and on the rate of progression of disease. Respiratory muscles are often involved in patients with proximal limb weakness, as in certain myopathies (Rosenow and Engel, 1978b; Braun et al., 1983; Mellies et al., 2005; Bi et al., 2021). Braun et al. (Braun et al., 1983) studied 53 patients with proximal myopathy (16 with lung disease). Hypercapnia was inversely related to VC (which, however, could be as high as 55% predicted), as well as to respiratory muscle strength (RMS, average of Pimax and Pemax). The latter relationship (an occlusive maneuver devoid of airflow and with negligible change in lung volume) reflected respiratory muscle weakness more than a decrease in VC which is affected by lung disease. Carbon dioxide retention did not occur until RMS was less than 50% predicted (Braun et al., 1983).

More recently, measurement of sniff nasal inspiratory pressures (SNIP) have been found to be better tolerated and reproducible (Ti and Fit Ting, 1999; Sarmento et al., 2021). Nasal pressure is measured in an occluded nostril as the patient sniffs through the opposite nasal passage and is closely associated with and easier to perform than Pimax (Laroche et al., 1988; Ti and Fit Ting, 1999) and FVC (Nicot et al., 2006) measurements. In a study of 61 patients with NMD and COPD, recording of esophageal pressure during a maximal sniff was found to be useful in assessing inspiratory muscle strength and easier to perform than the MIP (Sarmento et al., 2021). Many patients had normal sniff Pes despite low MIPs, suggesting that performing MIP was challenging because of dyspnea and weakness of facial muscles with difficulty in maintaining static pressures.

Transdiaphragmatic pressure (Pdi) can be recorded while breathing spontaneously or during maximal inspiratory maneuvers, such as a sniff (Perrin et al., 2204; Yang and Finkel, 2010). It can also be measured during magnetic stimulation of the phrenic nerve and has the advantage of greater accuracy for measuring diaphragm strength, especially in individuals with airflow limitation in whom airway opening pressure or SNIP would not accurately reflect esophageal pressure because of reduced lung elastic recoil and gas decompression. Measurement of Pdi and its role in the evaluation of diaphragmatic fatigue is described in detail in section 7.

### 10.3 Spirometry and Lung Volumes

Spirometry is essential in the diagnosis and management of pulmonary diseases and can be performed in children as young as 6 years old (Table 1) (Steier et al., 2007; Sharma, 2009; Caruso et al., 2015; Chiang et al., 2018). It facilitates assessment of functional capacity, following the course of illness, and response to management.

**TABLE 1:** Respiratory function tests commonly used to evaluate individuals with neuromuscular disorders.

Lung Volumes
Total lung capacity (TLC)
Residual volume (RV)
Spirometry
Peak flow rate (PFR)
Cough peak flow (CPF)
Forced vital capacity (FVC) (preferably in seated and supine positions)
Forced expiratory volume in 1 s (FEV_1_)
Maximum insufflation capacity (MIC)
Respiratory muscle strength
Maximal expiratory pressure (MEP) (preferably in seated and supine positions)
Maximal inspiratory pressure (MIP) (preferably in seated and supine positions)
Sniff test (SNIP)
Gas exchange
Oxyhemoglobin saturation by pulse oximetry
Capnography: end-tidal CO_2_ (PetCO2) measurement
Arterial or venous blood gas profile

Modified from (Sharma, 2009).

**FIGURE 5:**
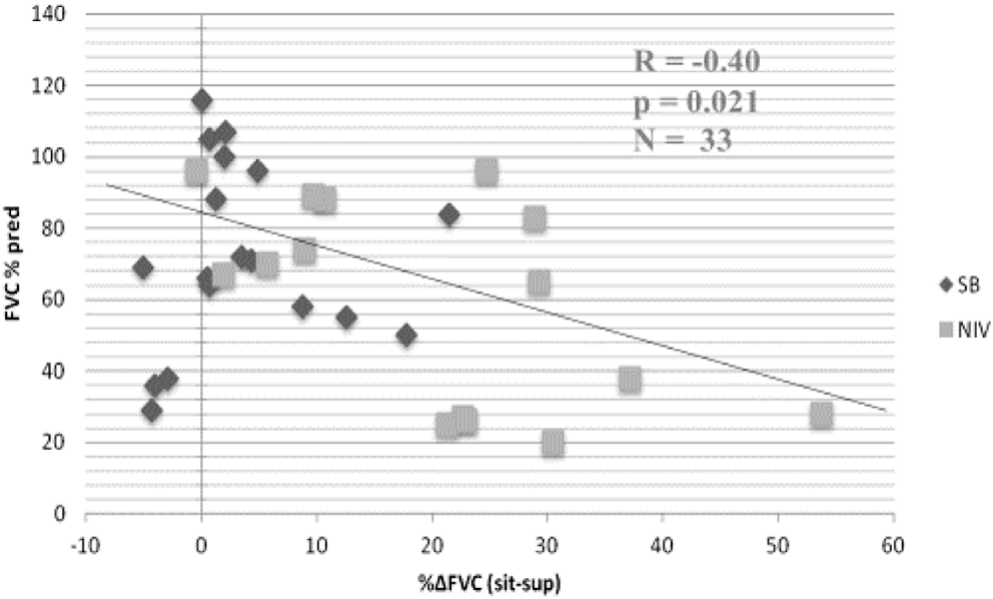
Relationship between % change in forced vital capacity (FVC) from seated (sit) to supine (sup) posture and baseline FVC in seated position in patients with neuromuscular disease (FVCsit, % predicted). Black diamonds, spontaneous breathing (SB); grey squares, noninvasive ventilation (NIV). Patients with greater than 20% decrease in FVC when supine required NIV. From (Chen et al., 2013).

**FIGURE 6:**
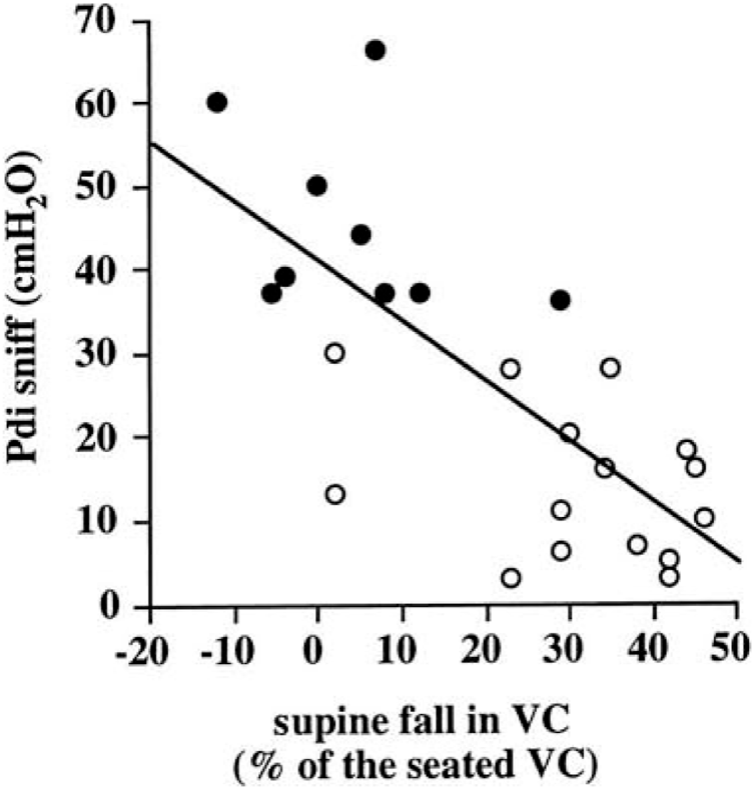
Relation between transdiaphragmatic sniff pressure (Pdi sniff) and supine fall in VC. Open symbols indicate patients with paradoxical diaphragmatic motion and closed symbols indicate patients without paradoxical diaphragmatic motion. From (Fromageot et al., 2001).

**FIGURE 7:**
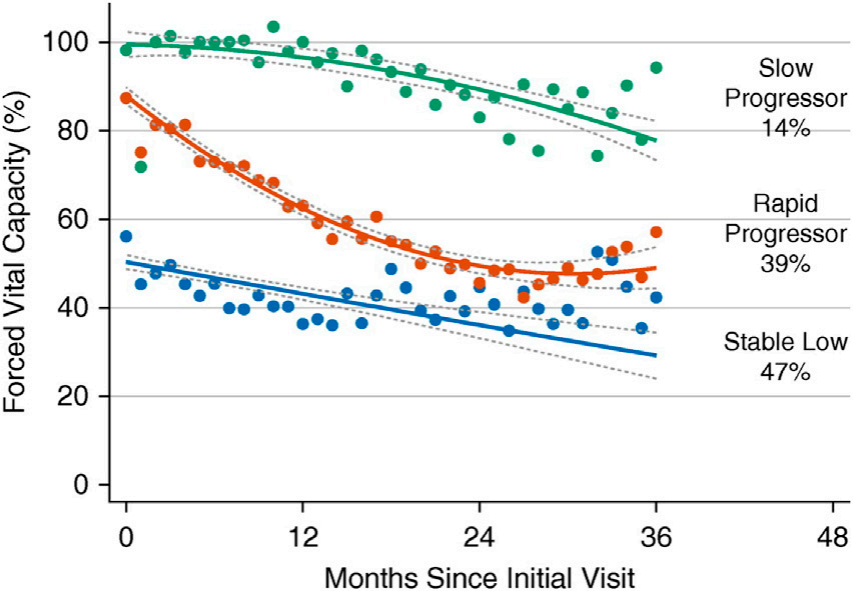
The Penn Comprehensive ALS Center cohort trajectories of forced vital capacity (FVC) percent predicted with 95% confidence intervals. Three groups of patients with amyotrophic lateral sclerosis (ALS) are indicated by slow progressors (green), rapid progressors (red), and stable low (blue). Percentages represent proportion of the cohort with the corresponding group number as the highest predicted posterior probability. From (Ackrivo et al., 2019a).

The forced expiratory volume in one second (FEV_1_) and FVC will show different rates of decline, depending on the course of illness. However, the ratio FEV1/FVC remains within normal range (80–100%). Using FEV1, respiratory health can be graded from mild to very severe: mild >70% predicted; moderate 60–69%; moderately severe 50–59%; severe ranging 35–49%; and very severe <35% (Sahni and Wolfe, 2018; Allen, 2010). Table 1 lists tests useful for evaluating respiratory function in patients with NMD and chest wall disorders. In a study of 60 patients with Duchenne muscular dystrophy, FVC and peak expiratory flow (PEF or Pemax) decreased linearly by 5 percent per year (Mayer et al., 2015). An FVC of <40% predicted was associated with a PaCO2 ≥45 mm Hg and a base excess of >4 mmol/L. Some have reported the value of determining the plateau VC which correlates with severity of Duchenne dystrophy (DMD) and risk of developing severe scoliosis (Welch et al., 2019). Following the VC plateau may indicate the best point to initiate air stacking to maximum lung insufflations (Benditt, 2019). Inspiratory muscle weakness is detected by a 20% or more reduction in VC upon assuming supine posture (Fromageot et al., 2001; Lechtzin et al., 2002; Prigent et al., 2012; Chen et al., 2013) and indicates need for assisted ventilation (Chen et al., 2013) (Figure 5). The reduction in lung volume also results in a decrease in maximal transdiaphragmatic sniff pressure (Pdimax sniff) (Figure 6). In general, assisted ventilation is indicated for patients whose VC has decreased below 1 L or 30% predicted (Steier et al., 2007; Sharma, 2009; Mayer et al., 2015; Chiang et al., 2018). Decline in FVC can be variable in patients with amyotrophic lateral sclerosis (ALS) (Ackrivo et al., 2019a), indicating phenotypes with differing prognostic implications (Elamin et al., 2015; Ackrivo et al., 2019b) (Figure 7).

Lung volume subdivisions can be measured using body plethysmography, gas dilution, or washout techniques (Table 1). If airflow limitation is suspected, plethysmography is the preferred method to avoid underestimating volume of trapped gas (Sharma, 2009; Mayer et al., 2015; Chiang et al., 2018). The method distinguishes air trapping from effects of obesity; both conditions result in reduction of expiratory reserve volume, while inspiratory capacity is reduced with air trapping but is increased with obesity. In advanced stages, preferential weakening of abdominal muscles with decrease in maximal expiratory pressure (Pemax) may explain the preservation or increase in residual volume. Spasticity of rib cage muscles prevents outward recoil of the chest wall from being reduced further helping to maintain FRC and preserve residual volume (RV). Patients may experience difficulty in performing panting maneuvers in the body box because of bulbar weakness. Excessive upper airway and cheek compliance may also result in underestimation of FRC and RV (Jaeger, 1982).

### 10.4 Measurement of Flow Limitation and Airway Resistance--Old and New Techniques

**FIGURE 8:**
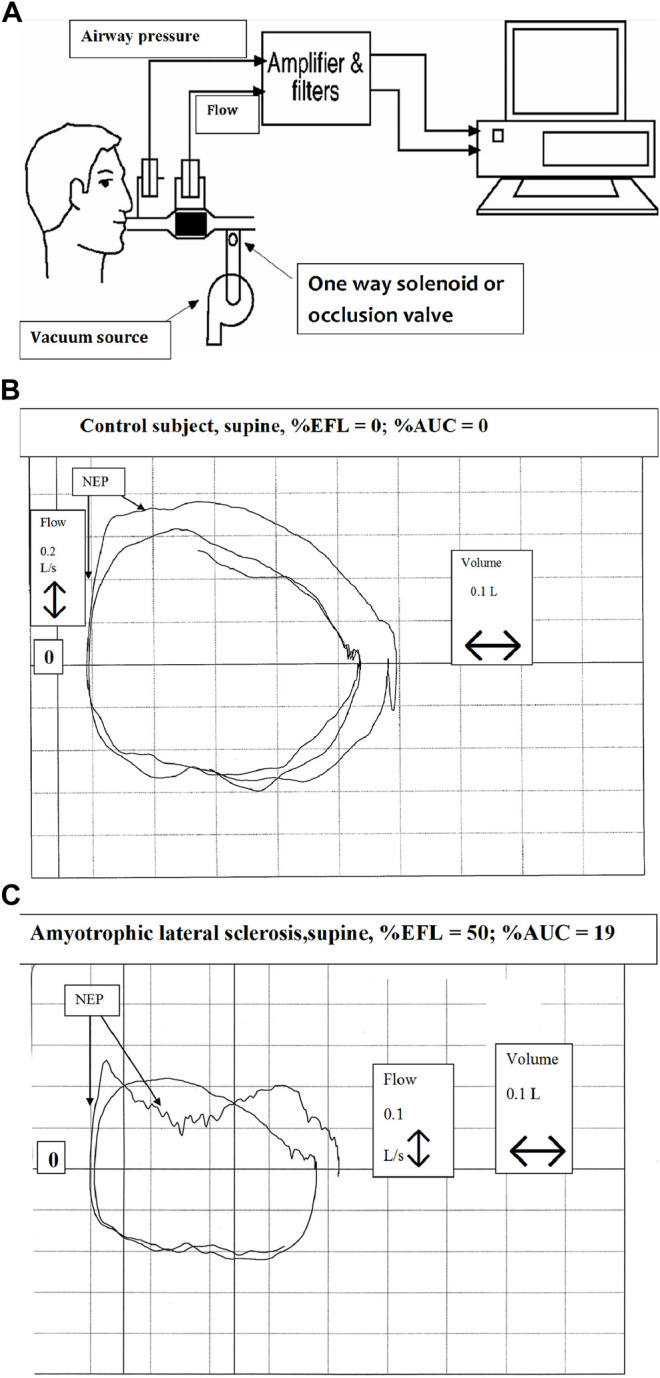
Technique of negative expiratory pressure (NEP) **(A)** Equipment setup for assessment of expiratory flow limitation during spontaneous breathing. NEP is applied at beginning of tidal expirartion **(B)** Tracings of airway pressure, volume and flow during quiet breathing in a healthy supine individual. Application of negative expiratory pressure at the onset of expiration is indicated by NEP **(C)** Tracings of airway pressure, volume and flow during quiet breathing in a supine individual with amyotrophic lateral sclerosis. Application of negative expiratory pressure at the onset of expiration is indicated by NEP. In C, the “dip” in expiratory flow during NEP indicates upper airway narrowing, suggestive of bulbar impairment. From (Baydur, 2013).

The presence of intrathoracic airflow limitation has been determined using the negative expiratory pressure (NEP) technique (Figure 8) in patients with NMDs (Baydur, 2013). It avoids technical challenges posed by spirometry such as lung history and inhomogeneity, forced expiratory maneuvers, and the speed of forced expiration, all influenced by viscoelastic properties of the lung and chest wall. NEP consists of comparing the tidal expiratory flow during application of gentle negative pressure (about −5 cm H_2_O) at the onset of expiration to the immediately preceding tidal expiratory flow. Expiratory flow should increase in healthy individuals while it changes little or not all in airflow limitation. Obese individuals or those with weakened bulbar musculature may exhibit transient decreases in tidal expiratory flow as upper airway soft tissues appose each other during application of NEP (Figure 8C). Such patients may be at risk for obstructive apneas during sleep as the upper airway may collapse during inspiratory efforts.

The forced oscillation technique (FOT) or impulse oscillometry (IOS) until recently was employed in infants and young children as a useful alternative to spirometry to assess airway mechanics. More recently, it has been increasingly used to assess respiratory resistance and impedance in older individuals with obstructive and restrictive disorders (Lappas et al., 2016). Even fewer studies have evaluated respiratory mechanics in individuals with neuromuscular disorders. Impedance measurements can be easily obtained as they require little cooperation and no forced respiratory maneuvers. In one study normal respiratory impedance (Xrs) characteristics were found, while respiratory resistance (Rrs) was somewhat higher than those found in normal subjects (Wesseling et al., 1992). Van Noord et al. (Van Noord et al., 1991) found that patients with kyphoscoliosis (TLC 41% based on arm span) exhibited an increase in total respiratory resistance and elastance.

### 10.5 Assessment of Control of Ventilation

The measurement of ventilatory drive by occlusion pressure recorded 100 msec after the onset of inspiratory effort (P_0.1_) is a noninvasive method that assesses central drive and has been measured in NMD(Whitelaw et al., 1975; Baydur, 1991). Several factors, however, can alter the relation between P0.1 and central drive, including presence of dynamic hyperinflation, expiratory muscle activity, chest wall distortion, respiratory muscle weakness, neuromuscular junction blockade, and the shape of the inspiratory pressure waveform (Whitelaw and Derenne, 1993). Central respiratory drive is blunted in patients with hypercapnic respiratory failure associated with NMD. In one study, NMD patients exhibited a hypercapnic drive response of only 30% that of normal subjects (García Río et al., 1994). The difference could be attributed to the reduced sensitivity of the chemoreceptors to chemical CO_2_.

### 10.6 Peak Expiratory and Cough Flows

Peak expiratory flow and cough flow (PEF and PCF, respectively) are used to assess ability to cough and clear airway secretions. Values of PEF less than 160 L/min are associated with increased risk for retained secretions, atelectasis and pneumonia (Bach and Saporito, 1996; Bach et al., 1997; Bach, 2017). Suarez and colleagues (Suárez et al., 2002) found differences between PEF and PCF to be 46%, 43% and 11% in normal subjects, patients with DMD and those with ALS, respectively, the last finding because of poor glottic closure. Determination of PEF and PCF can be applied to estimate respiratory muscle strength if maximal static mouth pressures cannot be performed (Suárez et al., 2002). Patients with PCF below 160 L/min benefit from mechanical in-exsufflation (Bach and Saporito, 1996; Bach et al., 1997; Polkey and Moxham, 2001; Suárez et al., 2002; Miller and Mayer, 2021).

### 10.7 Dyspnea and Sleep Quality

The reporting of dyspnea in patients with NMD may be related to sleep deprivation. Rault et al. (Rault et al., 2020) recently reported that a single night of sleep deprivation in a cohort of 20 healthy subjects reduced respiratory endurance during an inspiratory loading trial, accompanied by an increased sensation of dyspnea. More recently, using a questionnaire survey, the same group showed that the same individuals described a sensation of smothering more often than in a non-sleep-deprived control group (Rault et al., 2021). Thus, sleep deprivation modifies the sensation of dyspnea. While similar studies have yet to be done in individuals with NMD, increased respiratory elastance, and resistance, sleep deprivation should result in an increased sensation of breathlessness.

### 10.8 Oximetry and Capnography

Oximetry and capnography are useful to monitor gas exchange during studies of sleep disordered breathing. In patients with OSA (a chest wall disorder), oximetry may exhibit a sawtooth pattern during oxygen desaturation (Won et al., 2016) which represents cyclical variation in chemoreceptor response to fluctuations in ventilation or cardiac output (slow circulation time). Oximetry is more reliable when used in junction with capnography. While not as reliable as arterial blood gas analysis (because of changes in pH and CO_2_), the latter is invasive and increases work for the technologist and/or nursing. Capnography records CO_2_ tensions continuously using an end-tidal sensor or transcutaneous sensor. Disadvantages include expense, need for repeated calibration and susceptibility to external influences. A study simultaneously recording end-tidal and transcutaneous CO_2_ found that transcutaneous capnometry registered higher values of CO_2_ than the end-tidal method (Won et al., 2016). Using only end-tidal capnometry, the patient would not have been considered hypercapnic and thereby not offered assisted ventilation. Differences in recorded values should be considered when deciding to give a patient end-tidal or transcutaneous capnometry.

## 11 Recent and Future Developments

A number of recent molecular and physiologic approaches have demonstrated potential roles in further evaluation of respiratory function and treatment in NMD. Their continued role in clinical evaluation and management of neuromuscular-respiratory issues remains to be determined with more extended trials and clinical experience.

### 11.1 Gene Therapy

Recent developments in gene therapy have markedly improved care for NMDs (Markham et al., 2015; Harada et al., 2020; Oechsel and Cartwright, 2021; Paul et al., 2021). For instance, a new therapy called Poloxamer 188 NF improved respiratory function and measurements in dystrophic mice by targeting cardiomyocytes and improving intracellular calcium concentrations (Vincken et al., 1987). Plethysmographic measurements of dystrophic mice were similar to wild-type mice showing that the effects of muscular dystrophy could be minimized with the Poloxamer. Nusinersen, and onasemnogene aveparvovec (AVCS-101), a gene replacement therapy, have resulted in promising improvements in respiratory function and quality of life (Harada et al., 2020; Oechsel and Cartwright, 2021; Paul et al., 2021).

### 11.2 Electrical Stimulation of Muscles

In ALS reinnervation of denervated muscle fibers is crucial for preserving motor function as a means of compensating for motor neuron degeneration in long-term survivors. Diaphragm pacing has been advocated as a means of preserving respiratory muscle function, or at least, slowing its deterioration, but remains controversial (DiPALS, writing committee, DiPALS, Study Group Collaborators, 2015; Bermejo et al., 2016). Its mechanism was originally thought to be related to an increase in muscle fiber tone, thereby increasing its contractile properties.

Electrical stimulation of motor nerves leads to a reverse recruitment of motor units, in which larger fibers with less input resistance are activated before the smaller fibers. Recent studies in ALS have shown the opposite effect with an accelerated deterioration in respiratory function and increase in mortality (Bermejo et al., 2016). The RespistimALS group (Bermejo et al., 2016) reported that pacing in individuals with ALS failed to exhibit, over time, a significant increase in maximal amplitude of motor unit potentials (MUPs). These patients eventually required noninvasive ventilation and experienced decreased survival. By contrast, those who did not receive muscle stimulation showed a progressive increase in MUPs over time. Because efficient reinnervation leads to motor unit enlargement with an increase in muscle fiber content of surviving units, the authors concluded that the absence of increase in MUP amplitude over time in the active stimulation group supported the concept of a pacing-induced defective reinnervation.

In contrast to directly stimulating the muscle, the phrenic nerve can also be stimulated in cases of muscle deterioration to strengthen the diaphragm. However, the efficacy of this technique is disputed by some. In a study assessing the effects of phrenic nerve stimulation in 13 patients with myasthenia gravis, Mier et al. (Mier et al., 1992) demonstrated absence of change in diaphragmatic action potentials and even a reduction in 5 of the patients’ action potentials. A study testing phrenic nerve stimulation in humans with ALS was terminated early because of an excessive mortality rate and complications such as pneumothorax and acute respiratory failure occurring in the test group (Gonzalez-Bermejo et al., 2016).

### 11.3 Magnetic Stimulation of Respiratory Muscles

Contrary to electrical stimulation, magnetic stimulation is more of a diagnostic tool. Through different positions and placements of electrodes, the phrenic nerve can be stimulated by cervical, anterior pre-sternal and unilateral/bilateral anterolateral magnetic stimulation, and CMAP can be recorded (Man et al., 2004). These assessments indicate which parts of the diaphragm would benefit from rehabilitation and prognosticate the muscle’s recovery.

### 11.4 Imaging of Respiratory Muscles

Ultrasound imaging of the diaphragm is a non-invasive means of measuring diaphragm thickness and inspiratory thickening because an increase in diaphragm thickness leads to inspiratory strength (Roussos et al., 1979b; Boussuges et al., 2021). When applied by experienced users it provides reproducible results, with good inter- and intra-observer reliability (Cohn et al., 1997; Schepens et al., 2015). The thickness of the diaphragm can be determined in more than 85% of measurements, with a low coefficient of variation (0.09–0.14). Goligher et al. (Roussos et al., 1979b) assessed the validity of ultrasound in assessing diaphragm thickness and function in patients on mechanical ventilation. They found that only the right hemidiaphragm thickness is able to be measured, not the left, to help track the patient’s status while on mechanical ventilation unless the patent had a unilateral injury. Thickness of the right hemidiaphragm is also related to the degree of contractile activation of muscle during ventilation, so this technique can be used to assess diaphragm atrophy (Roussos et al., 1979b). The technique has the potential to be applied in NMD to assess changes in diaphragm thickness and motion over time, but requires skill and experience. It would be best applied in individuals who have not received assisted ventilation in order to monitor any progressive muscle loss from the NMD itself.

More recently kinematic analysis of the diaphragm from three-dimensional magnetic resonance images has been able to assess diaphragm mechanics (Mogalle et al., 2016) and is more sensitive than lung function testing in detecting weakness of the muscle; individuals with Pompé disease compensate for impaired diaphragm function with increase in chest wall movement.

### 11.5 Evaluation of Respiratory Mechanics During Mechanical Ventilation in NMD and Chest Wall Disorders

An interesting concept to consider is the phenomenon of airway opening pressure (AOP) during inspiration from FRC to total lung capacity (TLC) in individuals with restrictive respiratory disorders such as NMD and chest wall deformities receiving mechanical ventilation. As noted above, such patients may exhibit expiratory flow limitation (EFL) (Baydur, 2013). In such situations, it is possible to record the pressure-volume relationships to assess respiratory compliance and the AOP to facilitate adjustment of tidal volume and positive end-expiratory pressure (PEEP) and optimize gas exchange.

The question arises as to the relation of AOP to expiratory flow limitation (EFL): do they correspond? An analogy of this feature can be made with acute respiratory distress syndrome (ARDS). Using the airway occlusion technique, Guerin et al. (Guérin et al., 2020) showed that in semi-recumbent ARDS patients at PEEP 5 cm H_2_O, EFL and AOP did not occur simultaneously. While most patients with EFL exhibited an AOP, nearly half of patients with AOP did not have EFL. The additional tissue resistance measured at the end of inspiration was higher in patients with EFL than in those without EFL but did not differ amongst patients with and without AOP. Meanwhile, the interrupter resistance (of the conducting airways) did not differ between EFL and non-EFL patients, suggesting that EFL occurred in small airways. Thus, applying increasing levels of PEEP results in higher AOP without abolishing EFL. In addition, lung dynamic elastance was higher in FL than in non-FL patients and had a good accuracy for detecting EFL.

While the histopathologic features of ARDS are different from that of the microatelectatic changes seen with thoracic cage disorders, the lung mechanical properties of the latter conditions should be similar to that of ARDS with increases in respiratory resistance and elastance. If the curve depicting the lung transpulmonary pressure-volume relationship in a ventilated neuromuscular or scoliotic patient shown in Figure 3 were recorded at lower lung volumes, a discreet inflection point and its relationship to the presence or absence of EFL could be detected. Thus, application of PEEP would be expected to shift AOP towards higher airway pressures. In turn, determination of the location of AOP would optimize ventilator settings to improve gas exchange while avoiding volutrauma. It is not known how differences in lung mechanics between ARDS and NMD/chest wall disorder patients would influence the relation between EFL and AOP. This aspect of respiratory mechanics is a suggested opportunity for further study.

## 12 Opportunities for Future Research

### 12.1 Serum Biomarkers for Inflammation

COPD causes inflammation resulting in skeletal muscle dysfunction. COPD increases inflammatory factors such as IL-6, TNF-α, IL-8, and C-reactive protein, as well as an increase in the generation of reactive oxygen species (ROS) (Kim et al., 2008). The combination of inflammatory factors and ROS may be the cause of muscle wasting in COPD. Muscle atrophy from COPD can also severely affect inspiratory muscles, limiting their function. These events are similar in patients with NMDs depending on the etiologic origin of muscle weakness, so in both diseases, serum biomarkers can be used to noninvasively evaluate muscle breakdown and effects on the inspiratory muscles. Therefore, future research could evaluate the association between respiratory impairment and these biomarkers.

The neurodegenerative changes associated with NMD are, in some ways, similar to the myriad changes documented with COPD and aging (Kim et al., 2008; Dobrowolny et al., 2021). Neuromuscular junctions (NMJ) undergo functional, morphological, and molecular alterations during aging, resulting in a progressive decrease in skeletal muscle mass and strength (sarcopenia), changes common in NMD. In addition to the intrinsic myoneural changes inherent of NMDs, ROS homeostasis can contribute to changes in the neuromuscular junction morphology and stability, leading to reduction in fiber number and innervation. For example, Puig-Vilanova et al. (Puig-Vilanova et al., 2014) found that in the diaphragm of COPD patients, compared to control subjects, muscle-specific microRNA expression was downregulated, while histone acetyltransferases (HATs) and deacetylases and myocyte enhancer factor 2C protein levels were higher; by contrast, DNA methylation levels, muscle fiber types and sizes did not differ between patients and controls. The authors concluded that these epigenetic events act as adaptive mechanisms used to overcome the continuous inspiratory loads of the respiratory system in COPD. As respiratory muscles in NMDs are subject to mechanical and oxidative stresses similar to those observed in COPD, epigenetic events may also regulate respiratory muscle dysfunction and are a potential fertile area of investigation in this group of disorders.

### 12.2 Changes in Respiratory Pressures Influenced by Inspiratory Maneuvers

The updated guidelines by the ERS in 2019 (Laveneziana et al., 2019) do not provide much detail about performance of the SNIP test, limiting the instructions to: “the test is performed at FRC and the subject is instructed to sniff quickly and deeply”. Although considered a more physiological maneuver, an individual can perform a sniff test suboptimally when not appropriately instructed. In the evaluation of respiratory muscle strength, the diaphragmatic control performed during the SNIP test influences the inspiratory pressure and contractile properties of inspiratory muscles This occurs due to changes in the pattern of muscle recruitment, which change the force velocity characteristics of muscles. Benicio et al. instructed patients to perform a maximal effort starting from relaxed FRC according to ATS/ERS guidelines (Laveneziana et al., 2019). Participants were initially trained to breathe with a slow diaphragmatic breathing pattern, allowing bulging of the anterior abdominal wall (also known as ballistic contraction, diaphC). They were then instructed to inhale deeply through the nose, while simultaneously moving the abdominal wall outward (ballistic inspiratory maneuver). The main findings of this study showed that the maneuver with diaphC compared to without diaphC 1) significantly reduced the SNIP value, 2) reduced contraction times and electrical activity of accessory inspiratory muscles, and 3) decreased absolute values of maximum relaxation rate (*p* < 0.01), maximum rate of pressure development (MRPD). These findings confirmed that SNIP values diminish when a ballistic contraction of the diaphragm muscle is performed during a sniff maneuver. Thus, instructions on diaphC are recommended for specifically targeting diaphragm activity and better performance of the SNIP test.

### 12.3 Power Spectrum of EMG

Analysis of the power spectrum of EMG is a relatively old technique to assess muscle fatigue in patients during and after exercise with and without COPD and has not been employed in NMDs. Casabona et al. (Casabona et al., 2021) studied the possibility of using the power spectrum of EMG with non-fatigueable exercises to estimate muscle fiber composition in patients with COPD due to the different spectral content of the sEMG signal, which depends on the fiber type composition. The conclusion was that the power spectrum of COPD patients was at higher frequencies, which aligned with the severity of the disease. Even though this experiment was done on leg muscles, the same principle may be applied to the diaphragm and other inspiratory muscles by using surface electrodes, or better, esophageal electrodes situated at the cardia. Profiles of electromyographic power spectrum can be generated during repetitive maximal diaphragmatic contractions during deep breathing maneuvers. Future research could focus on this potential for detecting respiratory muscle fatigue before it affects VC, Pimax or Pdi.

### 12.4 Muscle Fiber Type

Changes in fiber type composition occur in NMDs (Glaser et al., 2018). New investigational approaches may elucidate how muscle fiber type specification occurs during disease conditions. For example, skeletal muscle cell culture from human pluripotent cell resources can provide a new instrument to study differentiation of human skeletal myocytes into myotubes with specific fiber types in culture (Hosoyama et al., 2014; Jiwlawat et al., 2017). Such studies could elucidate mechanisms involved in changes of fiber composition and ratio in the skeletal muscle of certain NMDs, and how they influence respiratory muscle kinematics and pathology. Such work carries the potential to create an *in vitro* model of contractile sarcomeric myofibrils for disease modeling and drug screening to study neuromuscular diseases.

## 13 Conclusion

Thoracic cage disorders are characterized by reduced lung volumes and respiratory compliance. Both upper and lower airway changes contribute to increase in airway resistance. While respiratory muscle weakness is a key feature that contributes to respiratory failure, central drive may be increased as characterized by increase in P0.1. Changes in respiratory mechanics contribute to elastic and resistive load compensation. Diaphragmatic braking action during expiration helps prevent further reduction in lung volume and atelectasis. Diagnostic studies focus on the evaluation of lung volumes and flows, respiratory muscle strength, ventilatory drive and recording of electromechanical dissociation of respiratory muscles. Ultrasound evaluation of diaphragmatic thickness and motion may prove useful in predicting respiratory muscle fatigue and failure. Mechanical ventilation affords an opportunity for further assessment of diaphragmatic function and airway properties such as expiratory flow limitation and airway opening pressure, which may provide guidance in applying appropriate ventilator settings. Finally, utilizing biochemical, genetic modeling and cell culture techniques have the potential to elucidate mechanisms of neuromuscular degeneration and the potential for discovering therapeutic approaches to halting or even stabilizing the loss of skeletal muscle, including respiratory muscles.

## Data Availability

The original contributions presented in the study are included in the article/Supplementary Materials, further inquiries can be directed to the corresponding author.
